# Factors associated with non-compliance with breastfeeding recommendation: a retrospective survey in hepatitis B virus-infected mothers who had taken Nucleos(t)ide analogs during pregnancy

**DOI:** 10.1186/s12884-021-04020-z

**Published:** 2021-08-12

**Authors:** Er-Mei Li, Li-Xin Xiao, Zhen Xu, Zhi-Shuo Mo, Jia-Qian Li, Yong-Yu Mei, Chao-Shuang Lin

**Affiliations:** 1 Department of Infectious Diseases, the Third Affiliated Hospital of Sun Yat-Sen University, 600 Tianhe Road, Guangdong Province 510630 Guangzhou, China; 2 Guangdong Provincial Key Laboratory of Malignant Tumor Epigenetics and Gene Regulation, Medical Research Center, Sun Yat-Sen Memorial Hospital, Sun Yat-Sen University, 510120 Guangzhou, China

**Keywords:** Chronic hepatitis B, nucleos(t)ide analogs, breastfeeding, patient’s compliance

## Abstract

**Background:**

We encourage Hepatitis B virus-infected mothers to breastfeed postpartum, even when continuing pregnancy category B nucleos(t)ide analogs (NAs) treatment. However, a large proportion of the Hepatitis B virus-infected mothers were noncompliant with this breastfeeding recommendation. This study aimed to investigate the factors associated with noncompliance with breastfeeding recommendation in Hepatitis B virus-infected mothers who had received NAs treatment during pregnancy.

**Methods:**

A total of 155 mothers with chronic hepatitis B receiving NAs treatment for preventing mother-to-child transmission during the late gestation period were included and divided into exclusive breastfeeding (n = 63), mixed feeding (n = 34), and artificial feeding (n = 58) groups according to the postpartum feeding methods. Independent variables associated with feeding methods were analyzed using logistic regression analysis.

**Results:**

Compared to the breastfeeding and mixed feeding groups, the artificial feeding group had significantly more multiparity, later postpartum timing of stopping NAs treatment, and a lower proportion of having knowledge of NAs medications (all P < 0.05). In addition, multivariable logistic regression analysis confirmed that multiparity, later postpartum timing of stopping NAs treatment, and lacking knowledge of medication were independent factors associated with noncompliance with breastfeeding recommendation.

**Conclusions:**

Hepatitis B virus-infected mothers who stopped NAs treatment at late postpartum period or had less knowledge of medication were more likely to be noncompliant with breastfeeding recommendation. Strengthening health education for participants taking NAs may be an important method to improve compliance with breastfeeding recommendation.

**Supplementary Information:**

The online version contains supplementary material available at 10.1186/s12884-021-04020-z.

## Background

Hepatitis B virus (HBV) infects more than 2 billion people worldwide [1]. According to the World Health Organization (WHO), 257 million people were estimated to be chronically infected with HBV in 2015 worldwide [2], including 65 million women of childbearing age [3]. Mother-to-child transmission (MTCT) is the major route of hepatitis B virus spread, accounting for nearly half of global chronic infections [4]. The active-passive immunization (hepatitis B vaccines plus hepatitis B immunoglobulin [HBIG]) can effectively prevent nearly 90 % of the MTCT of HBV [5]. However, a small portion of newborns still encounters the failure of active-passive immunoprophylaxis in preventing MTCT [6].

Clinical studies in recent years have confirmed that nucleos(t)ide analogs (NAs) treatment, such as tenofovir disoproxil fumarate (TDF) and telbivudine (LDT), in the second and third trimesters of pregnancy can effectively reduce the MTCT of HBV [7–10]. As a result, the guidelines by several liver disease associations, including the Asian Pacific Association for the Study of the Liver (APASL) [11], the European Association for the Study of the Liver (EASL) [12], the National Institute for Health and Care Excellence (NICE) [13] and the American Association for the Study of Liver Diseases (AASLD) [14] all recommend high viral load chronic hepatitis B (CHB) pregnant women at the immune tolerance period to receive pregnancy category B NAs (such as TDF and LDT [15]) during the second and third trimesters to reduce the MTCT rate.

Breastfeeding has many benefits for the mothers (such as postpartum weight management and reducing the risk of ovarian and breast cancer and type 2 diabetes) and infants (such as reducing the risk of respiratory, gastrointestinal, and ear infections in infancy) [16]. Currently, however, there is no consensus on whether HBV-infected mothers receiving pregnancy category B NAs treatment should breastfeed or not. In the WHO guidelines [17] and Asian-Pacific clinical practice guidelines [11], no clear instructions were given about breastfeeding by HBV-infected mothers with TDF treatment. In both 2017 EASL [12] and 2018 AASLD guidelines [14], breastfeeding is not contraindicated in HBsAg-positive women on TDF-based treatment or prophylaxis. The 2015 Chinese guideline by the Hepatology Branch of Chinese Medical Association [18] did not recommend breastfeeding for mothers who need to continue pregnancy category B medications postpartum. In the 2019 Chinese guidelines [19], breastfeeding is no longer prohibited for HBV-infected mothers receiving NAs for preventing MTCT during the gestation period, but breastfeeding is still not clearly recommended.

It has been reported that breastfed infants have extremely lower TDF exposure than those exposed in the fetuses or children receiving tenofovir treatment [20–22]. TDF and LDT belong to pregnancy Category B medications, and TDF has low potential toxicity in breastmilk. Therefore, we believe that breastfeeding should not be contraindicated in HBV-infected mothers on NAs treatment. In clinical practice, we recommend HBV-infected mothers to breastfeed postpartum, even when continuing pregnancy category B NAs treatment. We had previously conducted a prospective study on the safety of NA withdrawal in pregnant women with chronic hepatitis B in the immune tolerance period [23]. In the follow-up during the medication period, we conducted health education for these patients to advocate postpartum breastfeeding. However, it was found that a significant proportion of the HBV-infected mothers did not follow the breastfeeding recommendations and instead adopted artificial feeding. Therefore, the purpose of this study was to investigate the factors associated with noncompliance with breastfeeding recommendation in HBV-infected mothers who had received NAs treatment during pregnancy for preventing MTCT.

## Methods

### Study design and participants

This was a retrospective survey from January 2017 to August 2019, in the Department of Infectious Disease of the Third Affiliated Hospital of Sun Yat-sen University, Guangzhou, China.

**Fig. 1:**
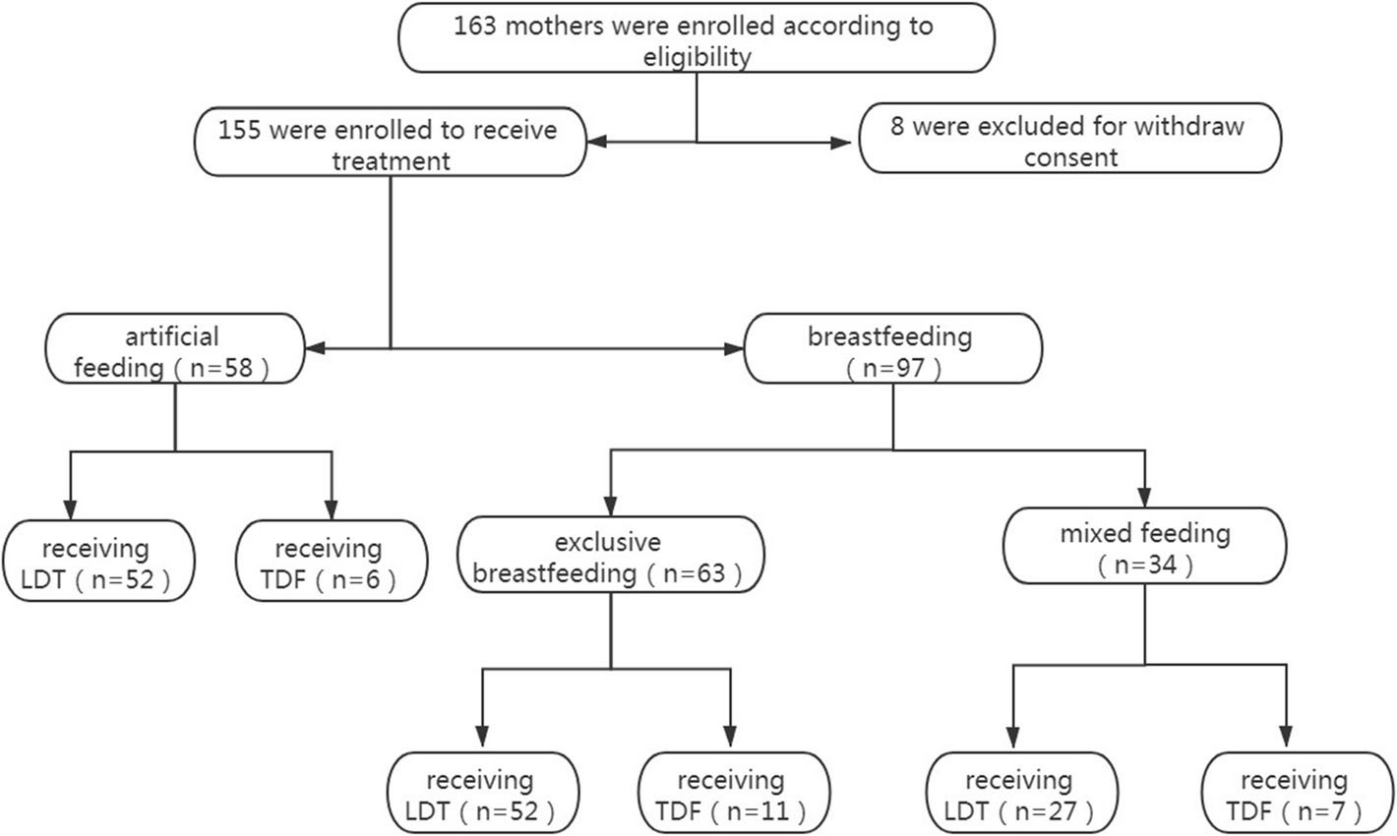
The flow chart of the study. LdT, telbivudine; TDF, tenofovir disoproxil fumarate

A total of 85 participants were retrospectively included from our previous study [1]. To expand the sample size of the study, another 78 HBV-infected women who met the inclusion criteria and had completed LDT/TDF treatment were included. A total of 163 participants were screened, and 8 of them withdrew their informed consent. Thus, 155 CHB participants with high viremia and in the immune-tolerant phase receiving NAs treatment for preventing MTCT during the gestation period were included. The flowchart of patient selection was shown in Fig. 1.

After the participants decided to receive medical treatment and signed the informed consent, health education was given during pregnancy, including the safety of LDT/TDF treatment and the safety of breastfeeding during the treatment. We ensured that the participants had sufficient knowledge about the following issues: 1). The safety of using this type of drug during pregnancy, 2). Whether it is necessary to continue antiviral treatment to block the mother-to-child transmission of HBV after delivery; 3) Whether it is possible to breastfeed when continuing antiviral therapy or stopping the drug during lactation. All participants were followed up for 12 months after delivery. The patients were also given health education on the risks and benefits of stopping the medication therapy immediately after delivery or at 6 weeks postpartum or more. After which, the participants would decide the timing of cessation of medication treatment by themselves.

The inclusion criteria were: (1) age between 18 and 45 years; (2) detectable HBsAg at the screening visit and at least 6 months prior; (3) positivity for serum HBeAg, HBV DNA level above 10^6^ IU/ml, and ALT level below the upper limit of normal (ULN; 40U/L). The exclusion criteria were: (1) coinfection with hepatitis A, C, D, or E virus or human immunodeficiency virus; (2) previous AVT for HBV infection (except for antivirals administered to prevent MTCT during a previous pregnancy and discontinued more than 6 months before the current pregnancy); (3) concurrent treatment with cytotoxic drugs, immune modulators, glucocorticoids or nephrotoxic drugs; (4) clinical signs of threatened miscarriage in early pregnancy; (5) evidence of hepatocellular carcinoma or cirrhosis; (6) evidence of fetal deformity by 3-dimensional ultrasound examination; (7) history of congenital malformation or congenital genetic disease in a previous pregnancy; (8) HBV infection of the husband (If the baby born to HBV-infected mother was still infected HBV even after receiving active-passive immunoprophylaxis, the possibility of father to child transmission can be ruled out).

This study was approved by the institutional review board of the Third Affiliated Hospital of Sun Yat-Sen University, Guangzhou (approval no. [2015]2-102). Written informed consent was obtained from each participant.

### Demographic and clinical characteristics

Participants’ demographic and clinical characteristics were collected from the medical records. Demographic and clinical characteristics were collected, mainly including age, education level, work status, fetal gender parity, delivery method, knowledge of medication (LDT/TDF), gestational age at the start of anti-viral therapy, postpartum timing of stopping NAs treatment, postpartum liver function and viral load, vaccination status, breastfeeding time, and whether breastfeeding with wounds. These data were categorized into categorical variables and continuous variables for statistical analysis.

### Data collection

All patients were followed up by telephone, and the information of all patients was recorded in the medical record. Artificial feeding was defined as using cow’s milk, goat’s milk, or other suitable milk substitutes including infant formula to feed the baby. A self-administered questionnaire (Supplementary material) was designed to collect the data including education level, working status, parity, delivery methods, the gestational age at the start of oral antiviral drugs, postpartum timing of stopping NAs treatment, postpartum liver function, postpartum viral load, infant gender, vaccination status, successful hepatitis b vaccination, breastfeeding time, breastfeeding with wounds, the decision of feeding method, child with unusually healthy issue, child with unusual height or weight, education on knowledge of medication (LDT/TDF) during prenatal care checkups.

Patients meeting the inclusion criteria would sign the informed consent form when taking NAs for preventing MTCT in the prenatal period, and they were formally included in the group during the return visit after delivery. Meanwhile, they were given questionnaires and telephone communication forms for long-term post-natal follow-up.

The patient’s knowledge of medication (LDT/TDF) was evaluated by a specialist doctor using a self-designed scale that consisted of several questions about mother-to-child transmission of LDT / TDF drugs.

### Statistical analysis

Continuous data were indicated with mean ± standard deviation (SD). For the comparisons between the two groups, the independent t-test or Mann-Whitney U test (if normality was not assumed) was used. Categorical data were indicated with number and percentage (%), and the distribution would be tested with the Chi-square test or Fisher’s exact test (if any expected value < = 5 was observed). One-way ANOVA was used for the means among groups (over 2 groups) and Fisher’s LSD test was used as post-hoc comparisons. Kruskal-Wallis would be used as a replacement if normality was not assumed. To investigate the associations between independent variables and feeding methods, the univariate and multivariable logistic regression models were used. The variables which reached P < 0.10 in the comparisons of mean differences were analyzed using logistic regression models. The significant variables (P < 0.05) in the multivariable model were recognized as factors associated with feeding methods. ROC analysis was used to investigate the diagnostic efficacy of continuous variables to dichotomous outcomes. A P < 0.05 was recognized as reaching the significance of each test, two-tailed. All analyses were performed using IBM SPSS Version 25 (SPSS Statistics V25, IBM Corporation, Somers, New York).

## Results

### Participant’s demographic and clinical characteristics

A total of 155 CHB mothers (mean age = 29.50 ± 3.55 years) receiving NAs treatment for preventing MTCT during the gestation period were included. Participant’s demographic and clinical characteristics were summarized in Table 1. NAs antiviral treatment included LDT (n = 131, 84.52 %) and TDF (n = 24, 15.48 %). The mean gestational age was 39.92 ± 2.41 weeks. The delivery methods included vaginal (n = 114, 73.55 %) and cesarean section deliveries (n = 41, 26.45 %).

**Table 1:** Clinical characteristics among different feeding methods

Parameters	N	Exclusive breastfeeding (n = 63)	Mixed feeding (n = 34)	Artificial feeding (n = 58)	All (n = 155)	P
Age, year	155	29.08 ± 3.30	27.88 ± 2.96	29.50 ± 3.55	28.97 ± 3.36	0.079
Educational level						0.233
Junior and senior high	31	8 (25.81 %)	6 (19.35 %)	17 (54.84 %)	-	
Undergraduate	108	47 (43.52 %)	25 (23.15 %)	36 (33.33 %)	-	
Graduate and above	16	8 (50.00 %)	3 (18.75 %)	5 (31.25 %)	-	
Work status						0.933
Unemployed	42	17 (40.48 %)	9 (21.43 %)	16 (38.10 %)	-	
Part-time or freelance	14	7 (50.00 %)	2 (14.29 %)	5 (35.71 %)	-	
Full-time	99	39 (39.39 %)	23 (23.23 %)	37 (37.37 %)	-	
^a^Parity						
Primiparous	108	53 (49.1 %)	23 (21.3 %)	32 (29.6 %)		< 0.001
Multiparous	42	8 (19.0 %)	8 (19.0 %)	26 (61.9 %)		
Delivery method						0.366
Vaginal	114	48 (42.11 %)	27 (23.68 %)	39 (34.21 %)	-	
Cesarean section	41	15 (36.59 %)	7 (17.07 %)	19 (46.34 %)	-	
Medication						0.361
LDT	131	52 (39.69 %)	27 (20.61 %)	52 (39.69 %)	-	
TDF	24	11 (45.83 %)	7 (29.17 %)	6 (25.00 %)	-	
Gestational age at start of anti-viral therapy	155	25.19 ± 4.17	24.18 ± 4.07	24.67 ± 2.96	24.77 ± 3.73	0.430
Postpartum timing of stopping NAs treatment						0.022
Delivery day	110	50 (45.45 %)	28 (25.45 %)	32 (29.09 %)	-	
1 month	3	1 (33.33 %)	2 (66.67 %)	0 (0.00 %)	-	
1.5 months	18	6 (33.33 %)	2 (11.11 %)	10 (55.56 %)	-	
2 months	1	1 (100.00 %)	0 (0.00 %)	0 (0.00 %)	-	
3 months	1	1 (100.00 %)	0 (0.00 %)	0 (0.00 %)	-	
6 months	1	0 (0.00 %)	0 (0.00 %)	1 (100.00 %)	-	
9 months	1	0 (0.00 %)	0 (0.00 %)	1 (100.00 %)	-	
Never	20	4 (20.00 %)	2 (10.00 %)	14 (70.00 %)	-	
Postpartum liver function						0.158
Normal	94	39 (41.49 %)	22 (23.40 %)	33 (35.11 %)	-	
Index rising	23	9 (39.13 %)	2 (8.70 %)	12 (52.17 %)	-	
Postpartum viral load						0.028
Normal	54	18 (33.33 %)	19 (35.19 %)	17 (31.48 %)	-	
Abnormal	79	35 (44.30 %)	12 (15.19 %)	32 (40.51 %)	-	
Infant gender						0.524
Male	78	34 (43.59 %)	18 (23.08 %)	26 (33.33 %)	-	
Female	76	28 (36.84 %)	16 (21.05 %)	32 (42.11 %)	-	
Vaccination on time						0.405
No	1	1 (100.00 %)	0 (0.00 %)	0 (0.00 %)	-	
Yes	154	62 (40.26 %)	34 (22.08 %)	58 (37.66 %)	-	
Successful hepatitis B vaccination						0.054
No	5	0 (0.00 %)	1 (20.00 %)	4 (80.00 %)	-	
Yes	142	59 (41.55 %)	30 (21.13 %)	53 (37.32 %)	-	
Breastfeeding months	-	9.16 ± 4.38	5.56 ± 3.64	-	7.92 ± 4.47	< 0.001
Breastfeeding with wounds^*^						0.314
No	57	38 (66.67 %)	19 (33.33 %)	0 (0.00 %)	-	
Yes	32	19 (59.38 %)	12 (37.50 %)	1 (3.13 %)	-	
Decision of feeding method						0.319
Both parents	85	36 (42.35 %)	20 (23.53 %)	29 (34.12 %)	-	
Physician	39	19 (48.72 %)	6 (15.38 %)	14 (35.90 %)	-	
Mother alone	31	8 (25.81 %)	8 (25.81 %)	15 (48.39 %)	-	
Infant birth body weight, kg	155	4.22 ± 1.65	4.02 ± 1.43	3.71 ± 1.38	3.99 ± 1.51	0.179
Infant birth body length, cm	155	48.94 ± 3.42	49.82 ± 2.39	49.40 ± 2.09	49.31 ± 2.76	0.315
Child with unusually healthy issue						0.791
No	143	59 (41.26 %)	31 (21.68 %)	53 (37.06 %)	-	
Yes	4	1 (25.00 %)	1 (25.00 %)	2 (50.00 %)	-	
Knowledge of medication (LDT/TDF)						< 0.001
No	32	4 (12.50 %)	6 (18.75 %)	22 (68.75 %)	-	
Yes	120	58 (48.33 %)	28 (23.33 %)	34 (28.33 %)	-	
Child with unusual height or weight						0.382
No	149	61 (40.94 %)	33 (22.15 %)	55 (36.91 %)	-	
Yes	3	1 (33.33 %)	0 (0.00 %)	2 (66.67 %)	-	

^a^Three mothers did not provide their parity, of which two were exclusively breastfeeding and one was mixed feeding; one of primiparous did not provide the feeding method, and one of the multiparous mother did not provide the feeding method

^*^“Breastfeeding with wounds” was defined as follows: 1) The mother’s nipples were chapped or damaged, causing the baby to directly contact with the mother’s blood during breastfeeding; or 2) The baby’s lips and mouth may be damaged which can directly contact with breast milk

According to the postpartum feeding methods, the participants were divided into three groups: exclusive breastfeeding (n = 63, 40.65 %), mixed feeding (n = 34, 21.94 %), and artificial feeding (n = 58, 37.41 %) groups. The majority of participants (n = 111, 71.61 %) had their first parity. It was found that participants with multiparity were more likely to use artificial feeding (P = 0.003). The later the postpartum timing of stopping NAs treatment, the greater the possibility of using artificial feeding (P = 0.022). Participants who had more knowledge of medication (LDT/TDF) were more likely to have breastfeeding (P < 0.001). The exclusive breastfeeding group had significantly longer breastfeeding months than the mixed feeding group (P < 0.001).

**Table:** 

**Parameters**	**N**	**Exclusive breastfeeding **	**Mixed feeding **	**Artificial feeding **	**P value**
^a^Parity					<0.001
Primiparous	108	53 (49.1%)	23 (21.3%)	32 (29.6%)	
Multiparous	42	8 (19.0%)	8 (19.0%)	26 (61.9%)	

^a^Three mothers did not provide their parity, of which two were exclusively breastfeeding and one was mixed feeding; one of primiparous did not provide the feeding method, and one of the multiparous mother did not provide the feeding method

## Participant’s clinical characteristics between groups with or without breastfeeding

Participants were further dichotomously divided into groups with or without breastfeeding (mixed feeding includes breastfeeding). As shown in Table 2, the significances were similar to Table 1. The artificial feeding group had significantly multiparity, later postpartum timing of stopping NAs treatment, and a lower level of knowledge of medication (all P < 0.05).

**Table 2:** Clinical characteristics between groups with or without breastfeeding

Parameters	N	Breast and mixed feeding (n = 97)	Artificial feeding (n = 58)	All (n = 155)	P
Age, year	-	28.66 ± 3.22	29.50 ± 3.55	28.97 ± 3.36	0.133
Educational level					0.080
Junior and senior high	31	14 (45.16 %)	17 (54.84 %)	-	
Undergraduate	108	72 (66.67 %)	36 (33.33 %)	-	
Graduate and above	16	11 (68.75 %)	5 (31.25 %)	-	
Work status					0.987
Unemployed	42	26 (61.90 %)	16 (38.10 %)	-	
Part-time or freelance	14	9 (64.29 %)	5 (35.71 %)	-	
Full-time	99	62 (62.63 %)	37 (37.37 %)	-	
Parity	-	1.20 ± 0.42	1.45 ± 0.50	1.29 ± 0.47	0.001
Gestational weeks	-	40.04 ± 2.50	39.72 ± 2.24	39.92 ± 2.41	0.429
Delivery method					0.169
Vaginal	114	75 (65.79 %)	39 (34.21 %)	-	
Cesarean section	41	22 (53.66 %)	19 (46.34 %)	-	
Medication					0.171
LDT	131	79 (60.31 %)	52 (39.69 %)	-	
TDF	24	18 (75.00 %)	6 (25.00 %)	-	
Gestational age at start of anti-viral therapy	-	24.84 ± 4.14	24.67 ± 2.96	24.77 ± 3.73	0.794
Postpartum timing of stopping NAs treatment					0.002
Delivery day	110	78 (70.91 %)	32 (29.09 %)	-	
1 month	3	3 (100.00 %)	0 (0.00 %)	-	
1.5 months	18	8 (44.44 %)	10 (55.56 %)	-	
2 months	1	1 (100.00 %)	0 (0.00 %)	-	
3 months	1	1 (100.00 %)	0 (0.00 %)	-	
6 months	1	0 (0.00 %)	1 (100.00 %)	-	
9 months	1	0 (0.00 %)	1 (100.00 %)	-	
Never	20	6 (30.00 %)	14 (70.00 %)	-	
Postpartum liver function					0.132
Normal	94	61 (64.89 %)	33 (35.11 %)	-	
Index rising	23	11 (47.83 %)	12 (52.17 %)	-	
Postpartum viral load					0.289
Normal	54	37 (68.52 %)	17 (31.48 %)	-	
Abnormal	79	47 (59.49 %)	32 (40.51 %)	-	
Infant gender					0.261
Male	78	52 (66.67 %)	26 (33.33 %)	-	
Female	76	44 (57.89 %)	32 (42.11 %)	-	
Vaccination on time					1.000
No	1	1 (100.00 %)	0 (0.00 %)	-	
Yes	154	96 (62.34 %)	58 (37.66 %)	-	
Successful hepatitis B vaccination					0.145
No	5	1 (20.00 %)	4 (80.00 %)	-	
Yes	142	89 (62.68 %)	53 (37.32 %)	-	
Breastfeeding months	-	7.92 ± 4.47	-	7.92 ± 4.47	-
Breastfeeding with wounds*					0.768
No	57	57 (100.00 %)	0 (0.00 %)	-	
Yes	32	31 (96.88 %)	1 (3.13 %)	-	
Decision of feeding method					0.363
Both parents	85	56 (65.88 %)	29 (34.12 %)	-	
Physician	39	25 (64.10 %)	14 (35.90 %)	-	
Mother alone	31	16 (51.61 %)	15 (48.39 %)	-	
Infant birth body weight, kg	-	4.15 ± 1.57	3.71 ± 1.38	3.99 ± 1.51	0.080
Infant birth body length, cm	-	49.26 ± 3.11	49.40 ± 2.09	49.31 ± 2.76	0.760
Child with unusually healthy issue					0.997
No	143	90 (62.94 %)	53 (37.06 %)	-	
Yes	4	2 (50.00 %)	2 (50.00 %)	-	
Knowledge of medication (LDT/TDF)					< 0.001
No	32	10 (31.25 %)	22 (68.75 %)	-	
Yes	120	86 (71.67 %)	34 (28.33 %)	-	
Child with unusual height or weight					0.651
No	149	94 (63.09 %)	55 (36.91 %)	-	
Yes	3	1 (33.33 %)	2 (66.67 %)	-	

*“Breastfeeding with wounds” was defined as follows: 1) The mother’s nipples were chapped or damaged, causing the baby to directly contact with the mother’s blood during breastfeeding; or 2) The baby’s lips and mouth may be damaged which can directly contact with breast milk

## Independent variables associated with feeding methods

To further investigate the independent variables associated with feeding methods (with or without breastfeeding), logistic regression analysis was performed. The variables reaching P < 0.10 in Table 2 were included in the univariate and multivariable logistic regression models, such as educational level, parity, postpartum timing of stopping NAs treatment, infant birth bodyweight, and knowledge of medication.

As shown in Table 3, the independent factors associated with feeding methods were parity, postpartum timing of stopping NAs treatment, and knowledge of medication (all P < 0.01). These results suggested that participants with multiparity, later postpartum timing of stopping NAs treatment, and less knowledge of medication were more likely to use artificial feeding.

**Table 3:** Associations between independent variables to groups with or without breastfeeding

	Univariate		Multivariable	
Parameters	OR (95%)	P	OR (95%)	P
Educational level		0.087		0.177
Junior and senior high	ref.	-	ref.	-
Undergraduate	0.41 (0.18 to 0.93)	0.032	0.43 (0.17 to 1.10)	0.077
Graduate and above	0.37 (0.10 to 1.34)	0.130	0.34 (0.07 to 1.65)	0.179
Parity	3.12 (1.54 to 6.33)	0.002	3.21 (1.42 to 7.23)	0.005
Postpartum timing of stopping NAs treatment, levels	1.30 (1.13 to 1.51)	<0.001	1.36 (1.15 to 1.62)	<0.001
Infant birth body weight, kg	0.81 (0.64 to 1.03)	0.083	0.82 (0.62 to 1.09)	0.174
Knowledge of medication (LDT/TDF)				
No	ref.	-	ref.	-
yes	0.18 (0.08 to 0.42)	<0.001	0.22 (0.09 to 0.56)	0.001

## Discussion

The subject of breastfeeding among HBV-positive mothers has attracted more and more attention. Previous studies demonstrate that breastfeeding by HBV-infected mothers is safe and does not increase the risk of MTCT if the newborns have received active-passive immunoprophylaxis [24, 25], including HBeAg^+^ CHB mothers [26]. Although a previous study has shown that HBsAg, HBeAg, and HBV DNA may be presented in breastmilk [27] but generally cannot enter the infant blood through the internal barrier of the intestinal mucosa. Only when mucosal permeability is increased due to complications or injuries, the virus can enter the infant’s blood [28]. Therefore, guidelines have suggested that breastfeeding should be encouraged for infants undergoing the standard passive-active immunoprophylaxis [5, 29, 30]. However, there is no consensus on whether HBV-infected mothers receiving pregnancy category B NAs treatment should breastfeed. The 2015 Chinese guideline [18] did not recommend breastfeeding for mothers who need to continue pregnancy category B medications postpartum.

Although the label of antiviral drugs does not recommend breastfeeding while taking these drugs, clinical studies support the safety of these drugs during breastfeeding [31, 32]. The TDF and LDT belong to pregnancy Category B medications, and TDF has low potential toxicity in breastmilk [20–22]. In addition, both 2017 EASL [12] and 2018 AASLD guidelines [14] suggest that breastfeeding is not contraindicated in HBsAg-positive women receiving TDF-based treatment. Therefore, we encourage HBV-infected mothers to breastfeed postpartum, even when continuing pregnancy category B NAs treatment. TDF and LDT can quickly and effectively reduce the HBV DNA viral load of HBV-infected mothers. In addition, these nephrotoxic drugs are also safe and do not increase the risk of fetal birth defects or other serious diseases [33, 34]. Compared with the general population, the current study observed a similar rate of birth defects among infants with exposure to LDT/TDF [9, 34]. However, a large proportion of the HBV-infected mothers did not follow the breastfeeding recommendation. In the current study, of the 155 pregnant CHB women receiving NAs treatment during the gestation period, only 40.65 % of cases underwent exclusive breastfeeding.

In this study, we investigated the factors associated with noncompliance with breastfeeding recommendation in HBV-infected mothers who had received NAs treatment during pregnancy. Our results showed that the artificial feeding group had significantly multiparity than the breastfeeding and mixed feeding groups and multivariable logistic regression analysis showed that multiparity was the independent factor associated with artificial feeding. This observation is inconsistent with the previous finding that multiparity children are more likely to be breastfed [35]. However, we did not survey the feeding habits of prior parity in those with multiple parities. Therefore, the clinical meaning of this phenomenon is limited.

Among the 110 cases of stopping NAs treatment at the delivery day in this study, 45.45 and 25.45 % of cases adopted exclusive breastfeeding and mixed feeding, respectively; only 29.02 % used artificial feeding. However, of the 20 continuing NAs treatment after delivery, 70.00 % of the cases used artificial feeding. On the other hand, among the 120 cases with the knowledge of medication (LDT/TDF), 71.67 % of cases adopted breastfeeding or mixed feeding, while 28.33 % of cases used artificial feeding. By contrast, in 32 cases without the knowledge of medication, 68.75 % of the cases used artificial feeding. In addition, multivariable logistic regression analysis confirmed that both postpartum timing of stopping NAs treatment and knowledge of medication were independent factors associated with noncompliance with breastfeeding recommendation.

The association between later postpartum timing of stopping NAs treatment and artificial feeding should be attributed to participants’ concern that the drugs remaining in breastmilk may have an adverse effect on breastfed infants. However, the previous study shows that breastfed infants have a blood TDF concentration of only 2 -4 % of maternal blood [21], so breastfed infants have lower TDF exposure than those in exposed fetuses [20, 21]. Recently, Hu et al. have compared the levels of TDF exposure in fetuses, breastfed infants, and children receiving tenofovir treatment. Their results reveal that the daily TDF dose ingested from breastmilk represented only 0.01–0.04 % of the proposed pediatric therapeutic daily dose for children receiving TDF treatment and 0.5–16 % of those exposed to the fetuses [22]. These findings suggest that TDF has low potential toxicity in breastmilk. It is worth mentioning that even healthcare workers may not have systematic and comprehensive knowledge about HBV MTCT [19]. Therefore, a health education leaflet that explains the low concentration of category B pregnancy medications LDT / TDF in breastmilk may help improve breastfeeding compliance of HBV-infected mothers receiving NAs treatment during pregnancy.

In the least Chinese guidelines for the prevention and control of mother-to-child transmission of hepatitis B virus (2019 edition) [36], breastfeeding is no longer prohibited for HBV-infected mothers receiving NAs treatment during pregnancy, but breastfeeding is still not clearly recommended. Our findings could provide a reference for revising the guidelines to recommend breastfeeding for HBV-infected mothers receiving pregnancy category B NAs treatment. However, since this was a retrospective, single-center study with relatively small sample size, evidence from a large prospective trial is required to recommend changes to the existing guidelines. In addition, we did not survey the feeding habits of prior parity in those with multiple parities. Moreover, we did not analyze health behaviors that are important confounding factors for breastfeeding willingness, such as maternal smoking and pre-pregnancy obesity. These limitations should be addressed in future studies.

## Conclusions

In summary, our findings suggested that HBV-infected mothers who stopped NA treatment at late postpartum period or had or had less knowledge of medication were more likely to noncompliance with breastfeeding recommendation. Strengthening health education may improve breastfeeding compliance.

## Supplementary Information

**Additional file 1**. Questionnaire for postpartum Breastfeeding in hepatitis B virus-infected mothers taking LDT/TDF during pregnancy

## Data Availability

All data generated or analysed during this study are included in this published article and its supplementary information file.

## Abbreviations

HBV: Hepatitis B virus
WHO: World Health Organization
CHB: Chronic hepatitis B HCCs: hepatocellular carcinomas
MTCT: Mother-to-child transmission
HBIG: Hepatitis B immunoglobulin
TDF: Tenofovir disoproxil fumarate
LDT: Telbivudine
AASLD: American Association for the Study of Liver Diseases
SD: Standard deviation
