# Supplementary material for: Factors associated with non-compliance with breastfeeding recommendation: a retrospective survey in hepatitis B virus-infected mothers who had taken Nucleos(t)ide analogs during pregnancy
Source: BMC Pregnancy Childbirth. 2021 Aug 12;21:551. doi: 10.1186/s12884-021-04020-z (PMC8359301; doi:10.1186/s12884-021-04020-z)
Supplement: Supplementary file 1 — Additional file 1. Questionnaire for postpartum Breastfeeding in hepatitis B virus-infected mothers taking LDT/TDF during pregnancy [file 12884_2021_4020_MOESM1_ESM.docx]

**Questionnaire for postpartum Breastfeeding in hepatitis B virus-infected mothers taking LDT/TDF during pregnancy**

1. Name___________ Telephone____________ Age: _________ years old;

2. Education level: ①Junior high school ②High school ③College ④Undergraduate ⑤Other ______________;

3. Working status: ①Housewife ②Part-time, freelance ③Full-time work

4. Parity: ①First parity ②Second parity ③Third parity ④ Other ______________;

5. Delivery methods: ① normal delivery ② cesarean section ③ forceps assisted delivery ④ other: ______________;

6. The gestational age at start of oral antiviral drugs: ____________ weeks

7. The time to stop antiviral drugs: ① The day of delivery; ② 42 days after delivery ③The drug has not been stopped ④ Other ______________

8. Postpartum liver function: ______________; postpartum HBVDNA: ______________;

9. Date of birth of baby: _________year_________month_________day; baby's gender: ①male ②female

10. The birth weight of the baby: __________Kg; the length of ___________cm; the number of gestational weeks at birth __________ gestational weeks

11. Whether the child was vaccinated with hepatitis B immune globulin and hepatitis B vaccine on time at birth: ① Yes ② No

12. Whether the infant is successfully vaccinated against hepatitis B: ①Yes ②No ③Not check yet.

13. Child's feeding method: ①exclusive breastfeeding ②mixed feeding ③artificial feeding ④other ______________

14. Reasons for not breastfeeding: ______________________________________________________

15. Who decides how to feed the child: ①Mom ②Dad ③Both parents ④Other family members ______________

16. Whether there is nipple chapped or ruptured lips of children during breastfeeding: ①Yes; ②No; ③I don’t know. if nipple chapped occurs, do you stop breastfeeding? ：①Yes; ② No;

17. Breastfeeding time: ______________ months

18. Is there any abnormality in the regular health examination of the baby? ①No ②Yes_______________________________

19. The child’s current height ___________cm; weight __________Kg

20. Have you learned the safety of drugs (TDF/LDT) for breastfeeding during the prenatal care checkups? ①Yes ②No
